# Corroborating Psychological Rehabilitation With Cardiac Rehabilitation to Optimize Recovery in Post Coronary Artery Bypass Graft Patient: A Case Report

**DOI:** 10.7759/cureus.28169

**Published:** 2022-08-19

**Authors:** Moli J Jain, Vishnu Vardhan, Vaishnavi Yadav

**Affiliations:** 1 Department of Cardio-Respiratory Physiotherapy, Ravi Nair Physiotherapy College, Datta Meghe Institute of Medical Sciences, Wardha, IND

**Keywords:** coronary artery bypass graft, depression, anxiety, psychological rehabilitation, cardiac rehabilitation

## Abstract

Besides various advancements in technologies, cardiac surgeries are associated with various pulmonary and psychological consequences. In this article, we describe the case of an elderly female patient who presented to the emergency unit with complaints of severe chest pain, breathlessness, and sweating. She was diagnosed with triple vessel disease and underwent coronary artery bypass graft (CABG) surgery. Post-operatively she was anxious, restless, and complained of pain and difficulty in breathing. For which a comprehensive tailor-made rehabilitation program was designed by the Cardio-Pulmonary Physiotherapist, which included cardiac rehabilitation along with psychological rehabilitation, which proved to be effective in substantial gains in both physical and mental wellbeing. This provides a pathway towards the treatment planning and aspects of patient problems that should be focused on, along with an effective day-wise protocol to improve patients’ symptoms using both physical and mental perspectives. The patient was assessed using various outcome measures, which revealed drastic changes in breathlessness, depression, anxiety, pulmonary capacities, and overall quality of life. We conclude that anchoring psychological rehabilitation to cardiac rehabilitation will provide effective management and improve the overall quality of life of such patients and healthcare burden.

## Introduction

Despite the numerous possibilities for managing coronary artery disease, coronary artery bypass grafting (CABG) is indeed an avenue with precise indications and good mid- and long-term results, which enables patients to experience therapeutic benefits and contributes to an increase in life expectancy and overall quality of life [1]. The CABG surgery presents satisfactory results; however, sternal pain, and dysfunctional breathing in the postoperative period, resulting in decreased cough efficiency due to fast and superficial breathing and can result in post pulmonary complications (PPCs) such as atelectasis [2].

Aside from that, mood disorders, weakness, worry, stress, and depression are common psychological repercussions, all of which are disabling and distressing [3]. Hyperventilation is a common reaction to emotional extremes such as fear, anxiety, or stress [4]. Patients exhibit feelings such as fear, anxiety, and worry as soon as they are informed of the procedure's necessity. Furthermore, a hospital stay is known to bring discomfort, tension, sadness, unease, boredom, pain exacerbation, and anxiety [5].

The prevalence and persistence of depression and anxiety in such patients can have a direct impact on involvement in cardiac rehabilitation and lifestyle modification programs highlighted an association with increased risk of morbidity in the short and longer term. Reduced breathing exercises that modify carbon dioxide tolerance have been shown to provide therapeutic advantages to patients suffering from anxiety and depression, according to studies [6]. Corroborating the findings of this study presented herein, the Buteyko breathing technique helps in reducing breathing to cause air hunger, which is equivalent to giving the body a small, controlled dose of symptoms, which can be a great tactic for overcoming the fear of the sensations that come with a full-blown panic attack and restoring normal breathing volume by resetting the respiratory centre [7]. 

Physiotherapists are crucial in the preparation and rehabilitation of patients who have had surgical procedures especially in the immediate post-operative period. Aside from possessing a vast arsenal of techniques, incorporating rehabilitation for psychological elements at this time can be crucial and will provide effective management and improve the overall quality of life.

## Case presentation

Patient information 

A 66-year-old female with a known case of hypertension from 20 years presented to the medical emergency unit with complaints of difficulty in breathing at rest, sweating, and retrosternal chest pain and tightness, which was subtle at the beginning and eventually evolved in nature. Following examination, ECG was done suggestive of unstable angina and incomplete right bundle branch block (RBBB), and first-line medical management was given. She has been advised admission to the Cardiac unit for angiography, which reveals coronary artery disease-triple vessel disease (CAD-TVD). Following a series of tests, she was diagnosed as a case of CAD-TVD, grade III right-sided medical renal disease, and type II diabetes mellitus (HbA1c - 6.9%). She had off-pump CABG surgery via median sternotomy using a saphenous vein graft. She was then shifted to CVTS ICU and referred for physiotherapy on a post-operative day one (POD).

A physiotherapist visits the patient in CVTS ICU on POD one; she was on six liters of oxygen support via a simple face mask. She was anxious, nervous, and restless and complained of difficulty in breathing and pain over the suture site. On inspection, the central line, femoral line, foley catheter, and retro sternal drains were in situ. On cardiovascular examination, pulse rate was 110 beats/minute and blood pressure of 118/77 mm Hg, and S1 and S2 sounds were heard during auscultation. On respiratory examination, there was an increased respiratory rate, 24 breaths/min with 98% oxygen saturation on six liters of oxygen per minute. During the examination, the chest wall movement was diminished, and so was the engagement of the auxiliary muscles of inspiration in a dysfunctional pattern. Auscultation revealed bilaterally reduced breath sounds with mild crepitation throughout the lungs.

Table 1 discusses the timeline of the current episode.

**Table 1:** Timeline of the events with their findings and treatment given. ECG: Electrocardiography, USG: Ultrasonography, CAD-TVD: Coronary artery disease- triple vessel disease, ADLs: Activities of daily living.

S. No.	Timeline	Consultation	Findings	Suggestions
1.	Day 1	Emergency department	ECG: Right bundle branch block and Unstable angina	Advice admission and angiography, USG, and blood investigations.
2.	Day 2	Cardiology OPD	Angiography: CAD-TVD	Advice CABG surgery
3.	Day 6	CVTS department	Underwent CABG surgery	Post-operative she was shifted to CVTS ICU.
4.	Day 7	Cardio-Respiratory Physiotherapy	Dyspnea, pain at the suture site, and difficulty in ADLs.	Post-operative cardiac rehabilitation program.
5.	Day 20	Discharge	Improved clinical outcomes, and functional independence	Home exercise program
6.	Day 37	Follow up	Better clinical outcomes and improved quality of life	Continuation of the Home exercise program and advice phase II cardiac rehabilitation.

Diagnostic assessment and interpretation

**Figure 1:**
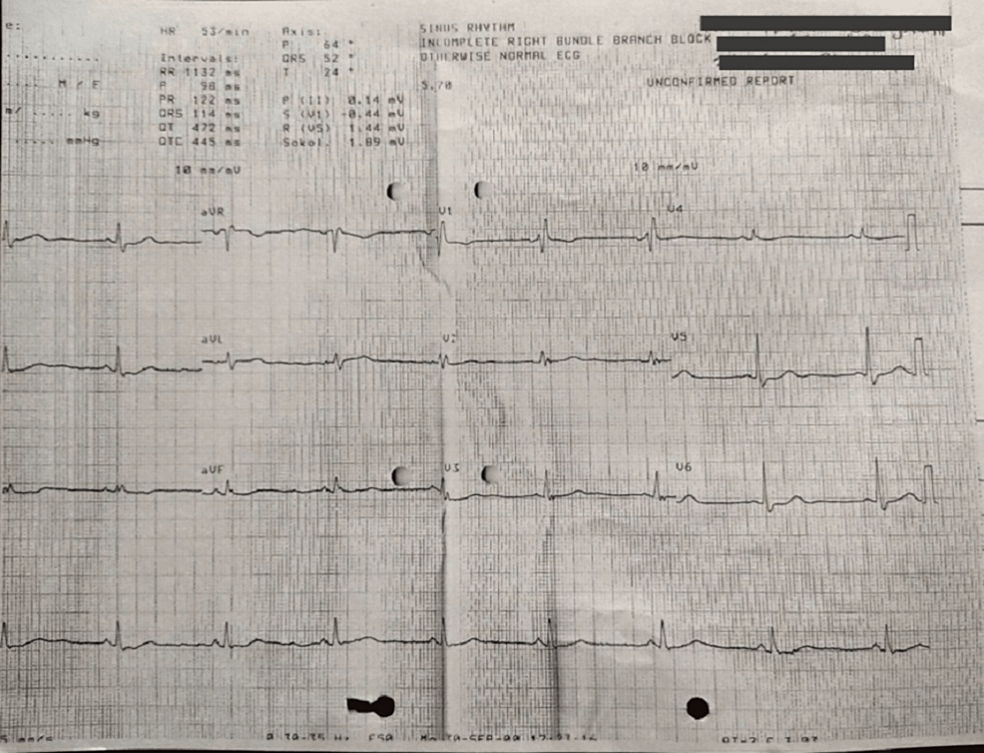
Pre-operative electrocardiography of the patient showed incomplete right bundle branch block with ST and T abnormality.

**Figure 2:**
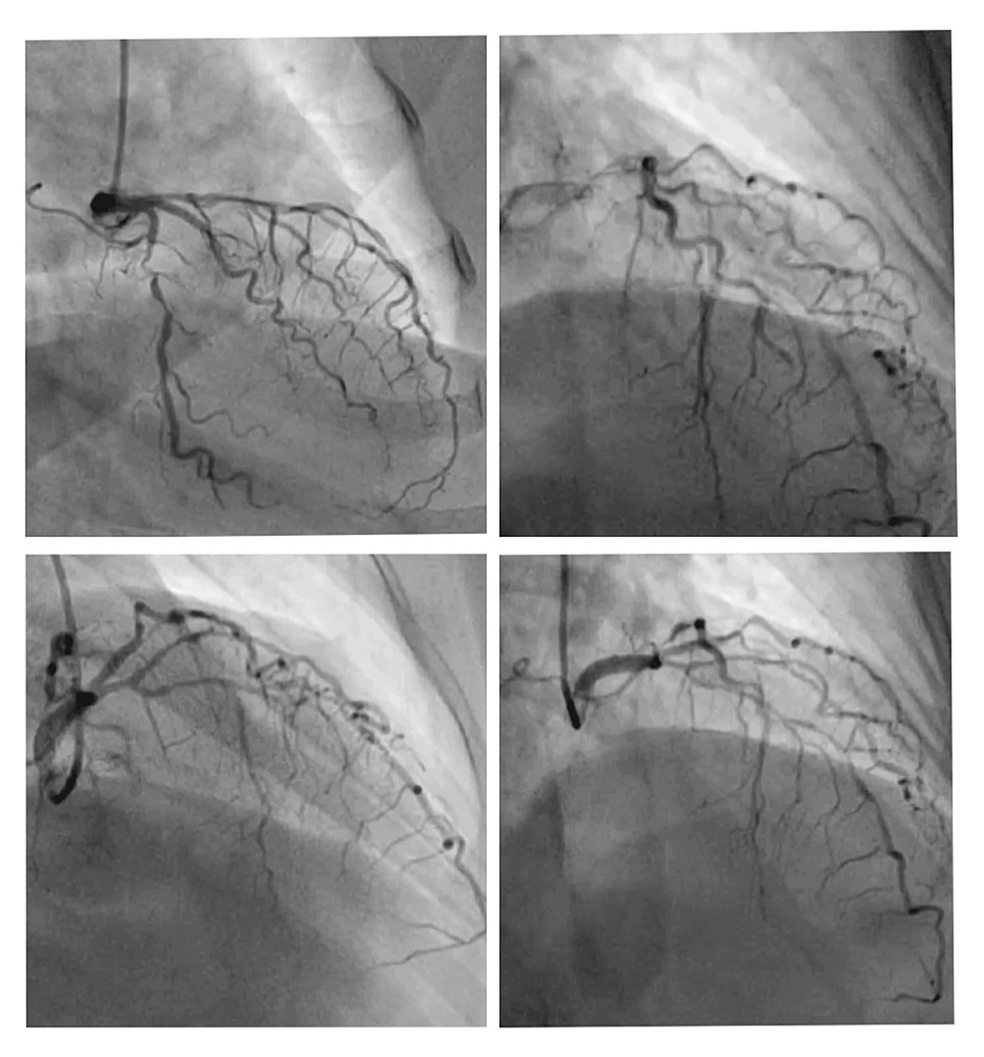
Pre-operative angiography reveals triple vessel disease.

**Figure 3:**
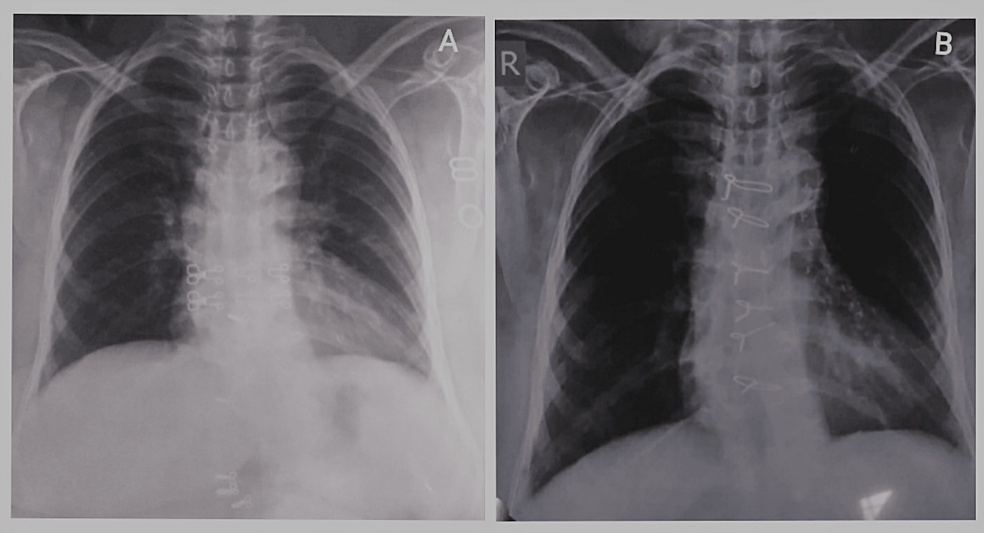
Pre-operative chest X-ray showed cardiomegaly (A) and in post-operative chest X-ray, sternal wires and cardiomegaly were noted (B).

ECG shows sinus rhythm, with incomplete right bundle branch block with ST and T abnormality (Figure 1). Coronary angiography findings revealed the left anterior descending artery (LAD) and its branches: type III vessel, mid 100% total occlusion, D1 proximal long 70%-80% stenosis. Circumflex: Non-dominant vessel, proximal segment 99% stenosis. Right coronary artery (RCA): Dominant vessel, proximal segment 80% stenosis, distal to the CRUX- 90% stenosis (Figure 2). Color doppler echocardiography shows type I diastolic dysfunction with preserved left ventricular ejection fraction of 55%. Ultrasonography findings showed the right kidney with increased cortical echo density with loss of cortico-medullary differentiation and parenchymal thinning suggestive of right-sided grade three medical renal disease. Pre-op and post-op chest radiography show cardiomegaly in pre-op and additional sternal wires in post-op (Figures 3A, 3B).

Therapeutic interventions

The patient was visited by a physiotherapist twice daily, monitoring and examination were done regularly, followed by rehabilitation sessions, and the outcomes were documented weekly. The patient's objective was to ease dyspnoea and let her return to her everyday tasks and spiritual activities (visiting the temple and praying). While keeping the patient's goals in mind, our goal was to alleviate dyspnoea, reduce discomfort and anxiety, improve breathing, promote relaxation, and improve overall functional status. Physiotherapeutic interventions were given to the patient for two weeks, the first week in CVTS ICU and the second week in the CVTS ward (Tables 2, 3).

**Table 2:** Summarizes the cardiac rehabilitation provided. TEE: Thoracic expansion exercise, IRV: Inspiratory reserve volume, FRC: Functional residual capacity, AROM: Active range of motion, ADLs: Activities of daily living, ACBT: Active cycle of breathing training.

Sr. No.	Intervention goals	Therapeutic Intervention	Treatment Regimen
1.	To prevent pulmonary, circulatory & integumentary complications post-surgery.	Manual positioning-half lying/semi-fowlers position was given initially; later upright sitting was given-air beds provided	Positioning was given after every two hours.
2.	To improve bed mobility and prevent prolonged immobilization	Monitored in bed transitional training and bedside mobilization given with binder	First three days: bedside sitting, chair sitting, and standing.
3.	To bring back to normal ADLs	Self-paced walking in 30 meters hallway	Begin on post-op day four, initially, five minutes, progressing up to 15-20 min, stair climbing on 13 to 14^th^ day
4.	To avoid strain over incision and drain site	Chest binders	Splinted coughing, Binder support during movements.
5.	To promote airway clearance	Acapella (green) ACBT	Ten reps x one set two times a day. Three to four cycles two times a day.
6.	To improve breathing patterns and respiratory rate	Deep breathing exercises: 1) Diaphragmatic breathing 2) Segmental breathing.	Initially, 10 reps x one set two times a day. Later ten reps x two sets three to four times a day.
7.	To improve lung volumes (IRV) and capacities (FRC)	1)TEE	Initially, 10 reps x one set two times a day. Later every two hours of interval
		2. Flow-oriented Incentive spirometer used.	
8.	To maintain joint integrity & mobility	AROM exercises of upper and lower limbs bilaterally.	Initially, 10 reps x one set two times a day. Later ten reps x two sets three to four times a day.
9.	To correct posture	Postural correction	Conscious correction of her posture by avoiding slouching by self-feedback and passive feedback from relatives whenever she was seen slouching.

**Table 3:** Summarizes the psychological rehabilitation provided.

S. NO	Intervention goals	Intervention	Regimen
1.	To provide awareness of the condition, and gain co-operation & consent of the patient and his family members	Patient and caregiver education and counseling about the exercise regimen and the importance of adherence to it.	Patient and caregivers were educated about the importance of positioning every two hourly, early ambulation, and adherence to an exercise regimen.
2.	Anxiety and depression	Buteyko breathing exercises, which consist of breath-holding and breath control exercises to promote shallow breathing patterns and nasal breathing to correct hyperventilation. Generalized relaxation exercise.	Initially, 10 reps x one set two times a day. Later ten reps x two sets three to four times a day.
3.	The feeling of unworthiness and useless-ness	Motivation and counselling of relatives and recreational activities.	One-to-one counselling session with patient and relatives. Recreational and spiritual activities.
4.	To retain the improvements throughout life	A counselling session for the patient and her relatives at the time of discharge.	Continuation of the Home exercise program and advice phase II cardiac rehabilitation.

Follow-up and outcome of the intervention

The outcome measures that were used to assess the progress of the patient on the first day of referral and the day of discharge are shown in Table 4. The patient was discharged with a set of exercise programs consisting of a self-monitoring technique for vitals and recognizing red flags, sternal precautions, and exercise guidelines with a daily activity tracker that the patients do at home to regain her strength and progress toward her goals. Along with this regimen, she was asked to perform breathing retraining and hygiene daily and to follow the relaxation, and dyspnoea relieving approaches whenever need be. Dietary requirements include a low-fat diet with normal salt intake and sugar as per diabetic status and to maintain ideal body weight. She was also advised to follow up after two weeks and can contact her telephonically for any doubts about the treatment or her condition. Good outcome of rehabilitation appears to be highly linked to patient satisfaction. She was very much satisfied with the prescribed protocol. She was hopeful and receptive during the treatment session, pleased with her development, she was willing to continue as long as advised.

**Table 4:** The values of outcome measures used to evaluate the progress of the patient.

Outcome Measure	Week 1	Week 2	Discharge
New York Heart Association scale	IV	III	II
Nijmegen Questionnaire	17	12	06
General Anxiety disorder-7 questionnaire	18	10	04
Patient health questionnaire-9	15	11	04
Arterial blood gas analysis	pO_2- _149 pCO_2-_30.40	pO_2-_129 pCO_2-_35.80	pO_2-_101 pCO_2-_43.50
Incentive spirometer	< 900 cc with 2- sec hold.	>900cc with 3-sec hold.	1200 cc with 3-sec hold.

## Discussion

Cardiac rehabilitation speeds up the recovery by preventing or resolving to post pulmonary complications and restoring to premorbid level. The physiotherapist is one of the professionals that spend the most time with the patients. As a result, it is proposed that time be better used by allowing specialists to clarify patients' doubts and lead them through the new scenarios they would confront. The high rates of anxiety and depression following heart surgery underline the importance of early detection, support, and management. Incorporating psychological intervention into an existing cardiac rehabilitation program will help in reducing pain, severe anxiety, hostility, and depression and thus improve quality of life as well [8].

To evaluate the efficacy of the treatment provided to the patient, outcome measures were used such as New York Heart Association to assess dyspnoea, Nijmegen Questionnaire was used for hyperventilation along with arterial blood gas analysis, General Anxiety disorder-7 and Patient health questionnaire-9 used for quantifying anxiety and depression respectively. All the scales/questionnaires were filled on the first day when the examination was done, on the day of discharge, and on the day of follow-up, the difference between the scores showed a whopping improvement on every scale.

The progress seen in the increasing walking distance motivated the patient to improve her respiratory capacity and stick to the protocol. The psychological consequences, the tendency of the patient to slouch, and forgetting to keep her posture straight, which might also be due to pain at the suture site, were the difficulties faced during the management of this patient.

## Conclusions

Although the patient did not fully recover during the rehabilitation program, the majority of the therapeutic goals were met, including improved breathing pattern, increased functional vital capacity, reduced pain, anxiety, and depression, improved chest expansion, and self-efficacy after two weeks of intensive cardiac and psychological intervention.
